# The cost of Medicare-funded medical and pharmaceutical services for mental disorders in children and adolescents in Australia

**DOI:** 10.1371/journal.pone.0249902

**Published:** 2021-04-09

**Authors:** Long Khanh-Dao Le, Sophy Shih, Scott Richards-Jones, Mary Lou Chatterton, Lidia Engel, Christopher Stevenson, David Lawrence, Genevieve Pepin, Cathrine Mihalopoulos

**Affiliations:** 1 School of Health and Social Development, Deakin University, Geelong, Australia; 2 Deakin Health Economics, Institute for Health Transformation, Deakin University, Geelong, Australia; 3 Graduate School of Education, The University of Western Australia, Perth, Australia; University of Bern, SWITZERLAND

## Abstract

**Objective:**

To examine the health care costs associated with mental disorders and subthreshold mental disorders within a nationally representative sample of children and adolescents in Australia.

**Method:**

Data were derived from the Young Minds Matter Survey (N = 6,310). Mental disorders were classified using the Diagnostic Interview Schedule for Children Version IV. Participant data were linked to administrative data on health care costs. Adjusted generalized linear regression models and two-part models were used to estimate mean differences in costs between those with a mental disorder or subthreshold disorder and those without.

**Results:**

Costs associated with health care attendances and medications were higher for children and adolescents with mental disorders and subthreshold mental disorders compared to those without a mental disorder. The additional population health care costs due to mental disorders amounted to AUD$234 million annually in children and adolescents, of which approximately 16% was attributed to out-of-pocket costs. Findings showed that those with subthreshold mental disorders or comorbid mental disorders have substantial additional costs of Medicare-funded medical and pharmaceutical services.

**Conclusion and implication:**

Mental disorders in children and adolescents are associated with significant health care costs. Further research is needed to ensure that this population is receiving effective and efficient care.

## Introduction

Mental disorders in children and adolescents have been associated with significant economic burden. The average annual cost per child (including the direct health care, direct non-medical, and indirect costs (usually defined as productivity losses)) have been estimated at €1,735 for conduct disorder (CCD), €1,782 for major depressive disorder (MDD), €1,077 for anxiety disorder and €781 for Attention-Deficit/Hyperactivity Disorder (ADHD) in Europe [1]. The annual cost of sub-threshold and clinical mental disorders (including both direct and indirect costs) was 4.7 and 9.2 times greater than the lifetime cost of having no mental disorders [2]. Fatori et al. 2018 found that the annual health sector costs accounted for more than half of the national costs of sub-threshold and clinical mental disorders in 6–14 year olds followed by costs associated with school problems and parental loss of productivity [2]. Both UK and US research have shown that amongst children and adolescents, having a mental disorder was associated with higher annual health care costs, at least two times more than those without any disorder [3]. Unfortunately, there is no study available that provides a national picture of the annual costs of all child and adolescent mental disorders in Australia [3].

In Australia, findings from the Young Minds Matter Survey (YMM) have shown that nearly one in seven (13.9%) 4–17 year-olds were assessed as having a mental disorder in the previous 12 months (measured by the Diagnostic Interview Schedule for Children Version IV) in 2013–2014 [4]. Males were more likely than females to have a mental disorder (16.3% compared with 11.5%). The most common mental disorder identified was Attention-Deficit/Hyperactivity Disorder (ADHD) (7.4%), followed by anxiety disorders (AnxD) (6.9%), major depressive disorder (MDD) (2.8%) and conduct disorder (CD) (2.1%). Of those who met the criteria for any mental disorder, nearly one-third (30.0%) had two or more mental disorders. YMM also collected information on health care resource use in children and adolescents in two ways. Firstly, via a resource use questionnaire (RUQ) that included the broad categories of health service use (including primary and specialist services, hospitalisation and medications), criminal/justice services, and online services. Secondly, participants were asked to provide consent to access their Medicare and Pharmaceutical Benefits Scheme data [5,6]. Medicare and Pharmaceutical Benefits Scheme refer to the universal federally funded programs for medical and allied health services, diagnostics and pathology (Medicare) and pharmaceuticals (Pharmaceutical Benefit Scheme).

Cost of illness studies are useful to estimate economic burden of disease and are gaining significant attention of policy-makers [7]. Previous systematic reviews indicated a lack of evidence to estimate the cost associated with mental disorders compared to those without mental disorders in children and adolescents such as depression [8] or anxiety disorders [9]. For depression, previous evidence showed that the total direct costs of those with depression were 179% higher than those without depression and the ratio decreased with age [8]. The study found that the highest levels of difference in cost of treating mental disorders in adolescence could have been explained by the provision of more resource-intensive treatment at younger ages [8]. While information collected through the RUQ provided important indicators of the economic burden associated with mental health conditions in Australian children and adolescents, responses in the RUQ may be subject to recall bias [10]. One advantage of Medicare and Pharmaceutical Benefits Scheme data is that it is complete (at least for services that are subsidised by the Commonwealth) and is not subject to recall bias—a major limitation of RUQs [10]. Every Australian citizen, permanent resident and some temporary residents are eligible for subsidised health care under the Medicare and Pharmaceutical Benefits Scheme schemes. Additionally, Medicare and Pharmaceutical Benefits Scheme data provides more detailed information on costs since both the government reimbursement of each occasion of service along with the actual out of pocket fees for the service are collected.

The aim of the current paper is to determine the annual average direct health care costs of mental disorders and subthreshold mental disorders in children and adolescents by analysing the Medicare and Pharmaceutical Benefits Scheme data of children and adolescents participating in the YMM survey. The second aim was to provide an in-depth analysis of costs for specific mental health services and general health-related services. Finally, the study aimed to extrapolate the incremental costs due to mental disorders and subthreshold mental disorders in children and adolescents at the Australian population level.

## Methods

### Young Minds Matter survey

The Young Minds Matter survey was a national household survey conducted from May 2013 to April 2014 by the Telethon Kids Institute (The University of Western Australia) on behalf of the Australian Government Department of Health. The aims of the survey were to determine the prevalence of mental health problems and disorders, the impact of these problems and disorders, the services used for these mental health problems and disorders and the role of the education sector in providing services for children and adolescents with mental health problems and disorders. In addition, information on general health, education, family characteristics, basic demographics, and socio-economic characteristics were also collected.

The survey included a face-to-face diagnostic interview with 6,310 parents and carers of children and young people aged 4–17 years and a self-report questionnaire from 2,967 young people aged 11–17 years. The survey collected information on the 12-month prevalence of mental disorders. Further details on the methodology of the YMM survey can be found elsewhere [11].

### Classifications of mental disorder diagnoses

Mental disorders were assessed using the *Diagnostic Interview Schedule for Children Version IV* (DISC-IV) [12]. The DISC-IV includes questions regarding the symptomology of a mental disorder in the 12 months prior to the interview. The answers to these questions allowed the generation of the 12-month prevalence of mental health diagnoses. Further details of diagnosis have been reported elsewhere [11]. The following mental disorder diagnoses, derived from the DISC-IV in the YMM survey, were used in the present study:

- Major Depressive Disorder (MDD)
- Attention-deficit/hyperactivity disorder (ADHD)
- Conduct Disorder (CD)
- Anxiety Disorder (AnxD) including social phobia, separation anxiety disorder, generalised anxiety disorder and obsessive-compulsive disorder. It was assumed a diagnosis of any of the four forms of anxiety constituted a diagnosis of anxiety.

Additionally, a group of children and adolescents with subthreshold mental disorders were identified. This group comprised those who exhibited sufficient symptoms to be defined as having a subthreshold diagnosis (those who have half or more of the required minimum number of symptoms to meet diagnostic criteria) in AnxD, MDD, ADHD, CD and did not meet any diagnosis of the four specific mental disorders identified previously (i.e. MDD, ADHD, CD and AnxD) [11]. Finally, those without any mental disorders or subthreshold mental disorders were categorised as a no mental disorder group.

### Health care costs

Health care costs were estimated for health care attendances and prescription medication outside the hospital setting by using data from the national Medicare and Pharmaceutical Benefits Scheme [13]. Consent to access the claimed Medicare and Pharmaceutical Benefits Scheme data was requested from parents for individuals aged under 14 and directly from the young person for those aged 14 and over. This consent covered Medicare and Pharmaceutical Benefits Scheme services used in the 12 months prior to the survey interview date. Through Medicare, the Australian government subsidizes mainly non-hospital based medical care up to a pre-determined amount (usually 85% of the scheduled fee), with the remainder paid by the patient as out-of-pocket costs (OOP). The costs of prescription medications are subsidized by the Pharmaceutical Benefits Scheme, in which a comprehensive, pre-determined list of medications is subsidized up to a designated co-payment. The corresponding 12-month prevalence of Medicare and Pharmaceutical Benefits Scheme service use was extracted from the survey Medicare and Pharmaceutical Benefits Scheme datasets.

#### Mental health and general health related MBS service

Neither Medicare nor the Pharmaceutical Benefits Scheme collect any diagnostic information. As such, the intent of a health professional visit is not captured for many types of professional services. In the current study, we defined mental health related services by either selecting specific provider specialties (including clinical psychologist, psychiatrist trainee, psychiatrist, non-clinical psychologist, nurse for mental health, social worker, and occupational therapist for mental health) and specific mental health-related Medicare and Pharmaceutical Benefits Scheme items (including items claimed under the Better Access scheme introduced in November 2006 which also included General Practitioner (GP) specific services, including the provision and review of mental health care plans).

The remaining Medicare and Pharmaceutical Benefits Scheme claims were classified as general health related, even though they may have included a mental health component. The Bettering the Evaluation and Care of Health (BEACH) study has previously identified that over 80% of consultations with GPs where a mental health related issue was discussed are not billed as mental health specific items [14]. The BEACH study has estimated that across all age groups approximately 12% of consultations with GPs in Australia are mental-health related, while less than 2% are identified as mental-health specific based on the Medicare Benefit Scheme item numbers [14].

#### Mental health and general health related PBS service

Mental health-related medications were defined as five selected medication groups classified in the Anatomical Therapeutic Chemical (ATC) Classification System by World Health Organization (WHO) [15] and used by the Australian Institute of Health and Welfare (AIHW). These medication groups included antipsychotics (code N05A), anxiolytics (code N05B), hypnotics and sedatives (code N05C), antidepressants (code N06A), and psychostimulants and nootropics (code N06B) [16]. In addition to these five mental health related medications, three other categories of medication were included in the YMM survey, including anti-hypertensives (C02A, C07A) and anti-convulsants (N03A). Anti-hypertensives include clonadine which is used in the treatment of ADHD and beta-blockers which are commonly used for anxiety in teenagers [17,18]. Given that very few children and adolescents receive treatment for hypertension compared with the off-label uses, it is most likely that children or adolescents who are prescribed these medications receive them for the treatment of mental disorders. Similarly, anticonvulsants are commonly used as mood stabilisers in people with bipolar disorder. The remaining medications utilised by study participants were considered as general health related.

### Statistical analyses

Statistical analyses were conducted using Stata 15. Population weights were developed to incorporate the characteristics of people who consented to Medicare and Pharmaceutical Benefits Scheme data to reflect the general population. These weights were developed in addition to the variables that are used in the YMM survey general population weights. Descriptive statistics were estimated for the mean (and 95% confidence interval) of mental health and general health related service uses and economic costs of healthcare services and pharmaceuticals by relevant comparison groups of mental disorder status. A generalized linear regression model (GLM) was used to estimate the mean differences in costs between groups (subthreshold/mental disorders vs. no mental disorders) adjusted for child gender, age, country of birth and socio-economic status (measured by the Index of Relative Socio-economic Disadvantage (IRSD) quintile) of the families. Population cost estimates were calculated by multiplying adjusted mean differences by the estimated population size, as reported in Australian 2019 population size statistics [19]. A two-part model was used to detect whether there was a difference in the probability of incurring any costs and the difference in costs of those who used services. The first part was a logistic regression model for the probability of incurring any costs and the second part was a GLM for costs incurred conditional on the cost being greater than zero. As recommended by the International Society for Pharmacoeconomics and Outcome Research guidelines, a gamma error distribution with a logarithmic link function was used in the GLM to model the skewed distribution of the data [20]. Cost estimates are presented in Australian Dollars ($) for the year 2013/2014. Regarding the number of health care visits, a hurdle model was used to detect the differences between groups in those who used services. Hurdle regression models combine a binary model (e.g. logit) to predict zeros with a zero-truncated negative binomial model to predict non-zero counts [21]. In case of a large mass of zeros, the two-part and hurdle count models, has often improved the fit of the models and has been useful for better understanding of the results [21].

## Results

The total sample of the survey was 6,310 of which 76% consented to access to Medicare and Pharmaceutical Benefits Scheme data (S1 Fig). The proportion of children and adolescents who consented to access to Medicare and Pharmaceutical Benefits Scheme data and had any recorded service use was 86% for healthcare services funded under Medicare and 29% for pharmaceuticals under the Pharmaceutical Benefits Scheme. The demographics of the survey sample have been reported elsewhere [4,22]. In brief, the cohort had a similar proportion of girls (48.4%) and boys (51.6%) with mean age of 10.4 years. Most primary carers of children and adolescents in the cohort were born in Australia (70.1%), and had a diploma or higher degree (69.8%).

### Medicare Benefit Scheme cost differences by mental disorder status

**Fig 1:**
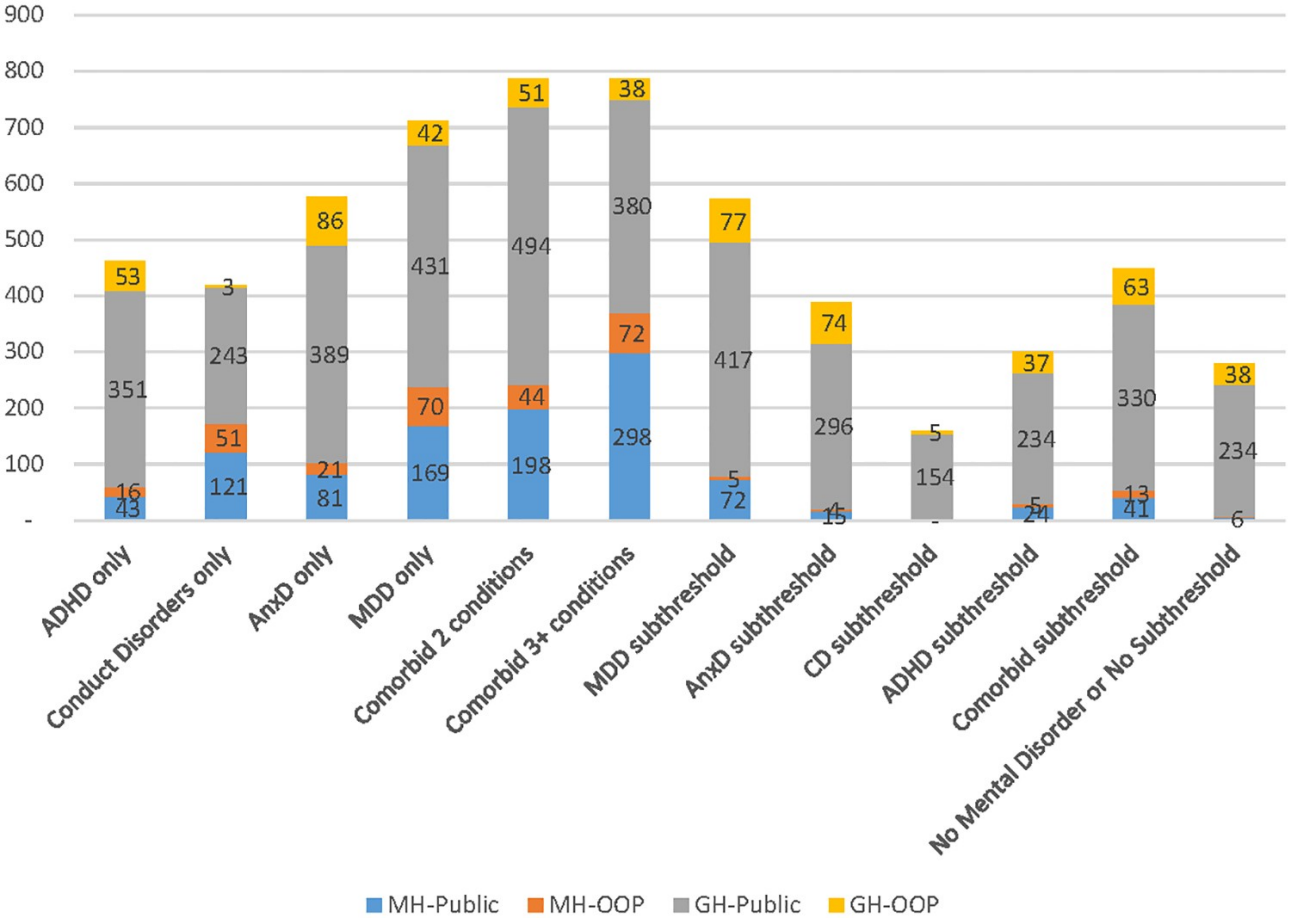
Average of total Medicare Benefit Scheme costs per person. MH-Public: Mental health related costs–benefit paid by the Government MH-OOP: Mental health related cost-out of pocket costs–out of pocket costs GH-Public: General health related costs–benefit paid by the Government GH-OOP: General health related cost–out of pocket costs ADHD: Attention-deficit/hyperactivity disorder, MDD: Major depressive disorder, AnxD Anxiety disorders, CD conduct disorder.

Fig 1 represents descriptive statistics for average annual Medicare Benefit Scheme mental health and general health-related health care service costs. Results showed that mental disorders were associated with higher annual average mental health specific costs. For example, the average annual mental health-related costs per person was found to be $7 for those without mental disorders versus $32 and $58 to $238 for those with subthreshold mental disorder and mental disorders, respectively. Among people with a single diagnosis, MDD was associated with the highest average annual mental health related costs ($238), followed by CD ($172), AnxD ($102) and ADHD ($58). Children and adolescents with comorbid mental disorders had higher average annual costs compared to those with single disorders (A$242 to A$370). Regarding subthreshold disorders, subthreshold MDD was associated with the highest average annual mental health and general health related costs, followed by subthreshold ADHD and comorbid subthreshold disorders. The adjusted differences in average annual costs between those with and without mental disorderswas statistically significant for both mental health and general health-related services, except for the average annual cost of both mental and general health-related services between those with CD or subthreshold MDD and without subthreshold or mental disorders.

**Fig 2:**
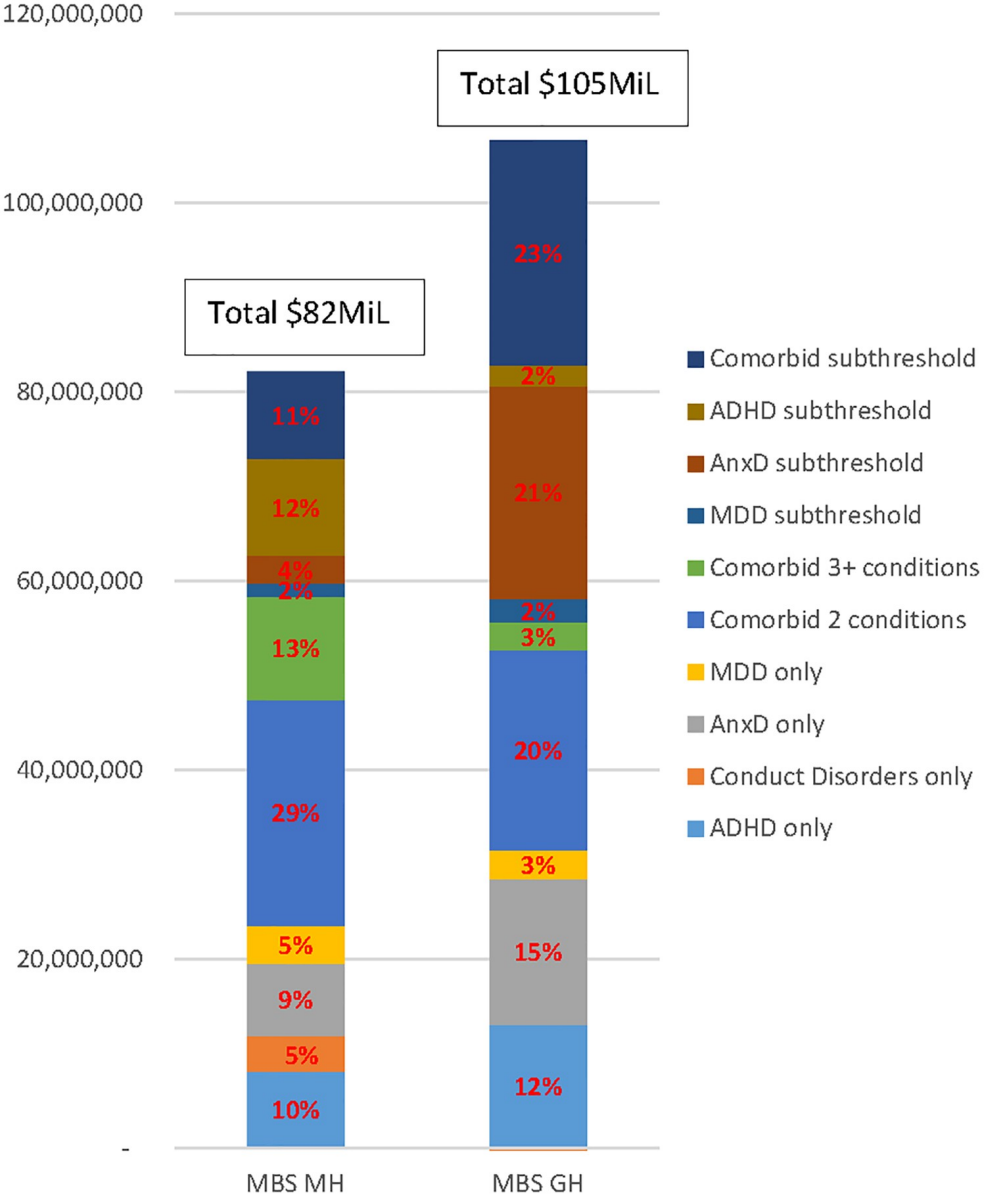
The additional health service costs due to mental disorders and subthreshold mental disorders. MBS MH: Mental health related cost–Medicare Benefit Scheme MBS GH: General health related cost–Medicare Benefit Scheme ADHD: Attention-deficit/hyperactivity disorder, MDD: Major depressive disorder, AnxD Anxiety disorders, CD conduct disorder.

Fig 2 shows the extrapolation of these findings to the entire Australian population, taking into account the proportions of children and adolescents in the relevant comparison groups. The estimated adjusted additional mental health and general health related cost to Medicare for healthcare utilisation associated with mental disorders and subthreshold mental disorders was $82M and $105M, respectively. The majority of these total additional costs were attributed to subthreshold mental disorders, especially for ADHD subthreshold, AnxD subthreshold and comorbid subthreshold mental disorders, followed by children/adolescents with two or three mental health diagnoses. With respect to single mental disorders, ADHD and AnxD contributed equally (9%–10%) to the total additional mental health related cost to Medicare, and similar proportions to general health related costs (12%–15%).

Table 1 presents results from the two-part models for mental and general health related costs of health care services. The first part of the model (logit model) indicates that those with a mental disorder or subthreshold mental disorder are more likely to have at least some costs. Children and adolescents with a mental disorder or a subthreshold mental disorder were more likely to use a mental health related service than those without a mental disorder except those with subthreshold MDD. Among those who had mental health related MBS expenditure, the hurdle model indicated children and adolescents with MDD or comorbid mental disorders or comorbid subthreshold mental disorders had significantly more mental health related health professional visits (two to three times) compared to those without a mental disorder. Similarly, these disorders together with AnxD or subthreshold ADHD were associated with significantly higher average cost than non-mental disorders in those who had mental health related MBS expenditure. Regarding general health care services, there is a significantly higher proportion using general health related services for those with MDD, 2 comorbid mental disorders or subthreshold comorbid mental disorders. For those who accessed general health related services, children and adolescents with a mental disorder or subthreshold mental disorder had a significantly greater number of health care visits (as well as average annual expenditure), except for those diagnosed with CD, three mental health conditions and subthreshold ADHD. Children and adolescents with ADHD, AnxD, two mental health conditions, or subthreshold mental disorders (except subthreshold ADHD) were found to have significantly greater medical service costs than children without a mental disorder.

**Table 1:** Results for count models for health care service and two-part models for total MBS costs.

Disorder categories	Mental Health specific services			General health related services		
	% using health service % (95% CI)	Number of visits for those who accessed services N (95% CI)	Average cost for those who accessed services $A (95% CI)	% using health service % (95% CI)	Number of visits for those who access services N (95% CI)	Average Cost for those who accessed services $A (95% CI)
No mental disorders	1.5 (1.0, 2.1)	2.7 (1.6, 3.8)	412 (277, 546)	84.2 (82.5, 86.0)	4.7 (4.4, 5.0)	322 (302, 341)
Any Mental Disorders	18.0 (15.0, 21.0)***	5.4 (4.5, 6.4)***	893 (749, 1037)***	90.0 (87.7, 92.4)***	7.2 (6.5, 8.0)***	503 (451, 555)***
ADHD	10.8 (6.4, 15.2)***	3.7 (1.9, 5.6)	622 (346, 898)	88.9 (84.2, 93.7)	6.0 (5.0, 7.1)*	455 (381, 530)**
Conduct disorders	17.4 (3.5, 31.3)*	7.1 (2.2, 12.0)	1005 (373, 1638)	83.3 (69.8, 96.7)	5.0 (2.2, 7.9)	303 (173, 432)
Anxiety related disorders	14.9 (9.7, 20.1)***	4.4 (2.8, 5.9)	667 (472, 861)*	88.2 (83.7, 92.7)	7.7 (6.0, 9.4)***	535 (401, 668)**
Major Depressive Disorders	14.3 (5.6, 23.0)**	6.1 (3.3, 8.9)*	1336 (628 2044)*	94.6 (89.4, 99.8)***	**8.5 (5.7, 11.3)** **	448 (315, 580)
Comorbid 2 conditions	27.5 (20.5, 34.6)***	6.5 (5.0, 7.9)***	951 (744, 1159)***	92.7 (88.5, 97.0)***	8.5 (7.0, 10.0)***	590 (487, 693)***
Comorbid 3+ conditions	34.3 (17.5, 51.2)***	6.1 (3.5, 8.8)*	1226 (605, 1846)*	92.5 (84.3, 1.0)	6.7 (4.6, 8.8)	456 (316, 596)
Any Subthreshold disorders	5.3 (4.0, 6.5)***	4.7 (3.7, 5.8)**	677 (539, 815)**	85.1 (83.3, 86.9)	5.6 (5.2, 6.0)***	397 (365, 428)
Subthreshold MDD	15.6 (0, 31.6)	4.9 (0.8, 9.1)	507 (148, 866)	80.9 (64.1, 97.8)	10.2 (6.0, 14.4)**	569 (345, 792)*
Subthreshold AnxD	3.5 (1.9, 5.0)*	3.9 (2.6, 5.3)	558 (391, 725)	86.2 (83.0, 89.4)	6.0 (5.3, 6.7)***	419 (353, 485)**
Subthreshold CD	0	N/A	**N/A**	82.1 (65.7, 98.6)	3.1 (1.8, 4.4)*	208 (134, 282)**
Subthreshold ADHD	5.1 (3.1, 7.1)**	4.9 (2.7, 7.1)	727 (447, 1008)*	82.4 (79.5, 85.3)	4.7 (4.2, 5.3)	338 (306, 370)
Subthreshold Comorbid	7.7 (4.9, 10.5)***	**5.3 (3.5, 7.1)**	746 (516, 976)*	88.5 (85.0, 92.1)*	6.2 (5.4, 7.1)***	458 (387, 530)***

***, statistical significance at the 0.1% level;

**, statistical significance at the 1% level;

*, statistical significance at the 5% level. Statistically significant results were bold.

MDD: Major depressive disorder, AnxD: Anxiety disorders, CD: Conduct disorder, ADHD: Attention-deficit/hyperactivity disorder.

Regarding OOP costs, adolescents with MDD only or co-morbid 3+ disorders incurred the highest out of pocket costs for mental health related services, but not for other general health related services. In fact, of the mental health related MBS costs (both government and OOP costs), 29% of these costs were attributable to OOP costs.

Finally, the majority of children with mental disorders or subthreshold mental disorders were accessing mental health professions on average more than two to three times compared to those without a mental disorder in the prior 12 months (Table 1).

### Pharmaceutical Benefits Scheme cost differences by mental disorder status

**Fig 3:**
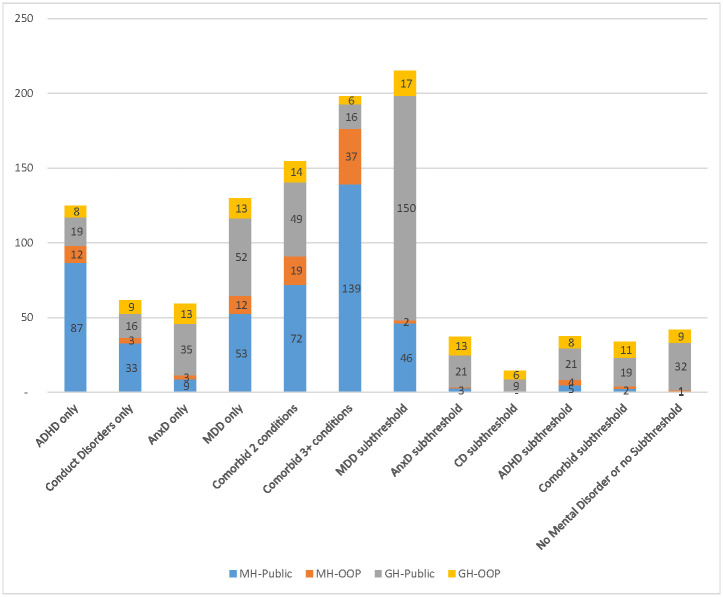
Total PBS cost per person. MH-Public: Mental health related costs–benefit paid by the Government MH-OOP: Mental health related cost–out of pocket costs GH-Public: General health related costs–benefit paid by the Government GH-OOP: General health related cost–out of pocket costs ADHD: Attention-deficit/hyperactivity disorder, MDD: Major depressive disorder, AnxD Anxiety disorders, CD conduct disorder.

Fig 3 presents descriptive statistics for the annual Pharmaceutical Benefits Scheme mental health and general health related costs. Similar to Medicare Benefits Scheme costs, unsurprisingly, mental disorders were associated with higher annual mean mental health related Pharmaceutical Benefits Scheme costs ($2 per person for those without mental disorders vs. $6 per person for those with subthreshold mental disorders vs. from $12 to $98 per person for those with mental disorders). In contrast to Medicare Benefits Scheme costs, ADHD was associated with the highest mental health related PBS cost per person ($98), followed by MDDs ($65), CDs ($36), and AnxDs ($12). Children and adolescents with comorbid mental disorders had approximately two times higher mental health related costs than those with a single mental disorder. The adjusted differences in costs of those with mental disorders and without mental disorders reached statistical significance only for mental health related Pharmaceutical Benefits Scheme costs. Regarding subthreshold disorders, MDD subthreshold was associated with the highest average cost of mental and general health related costs, however, there is no significant difference in costs between those with MDD subthreshold and those without. Mental and general health related costs were found to be similar between subthreshold ADHD, subthreshold AnxD and comorbid subthreshold mental disorders, and these costs were statistically significantly higher than those without a mental disorder or subthreshold symptoms.

**Fig 4:**
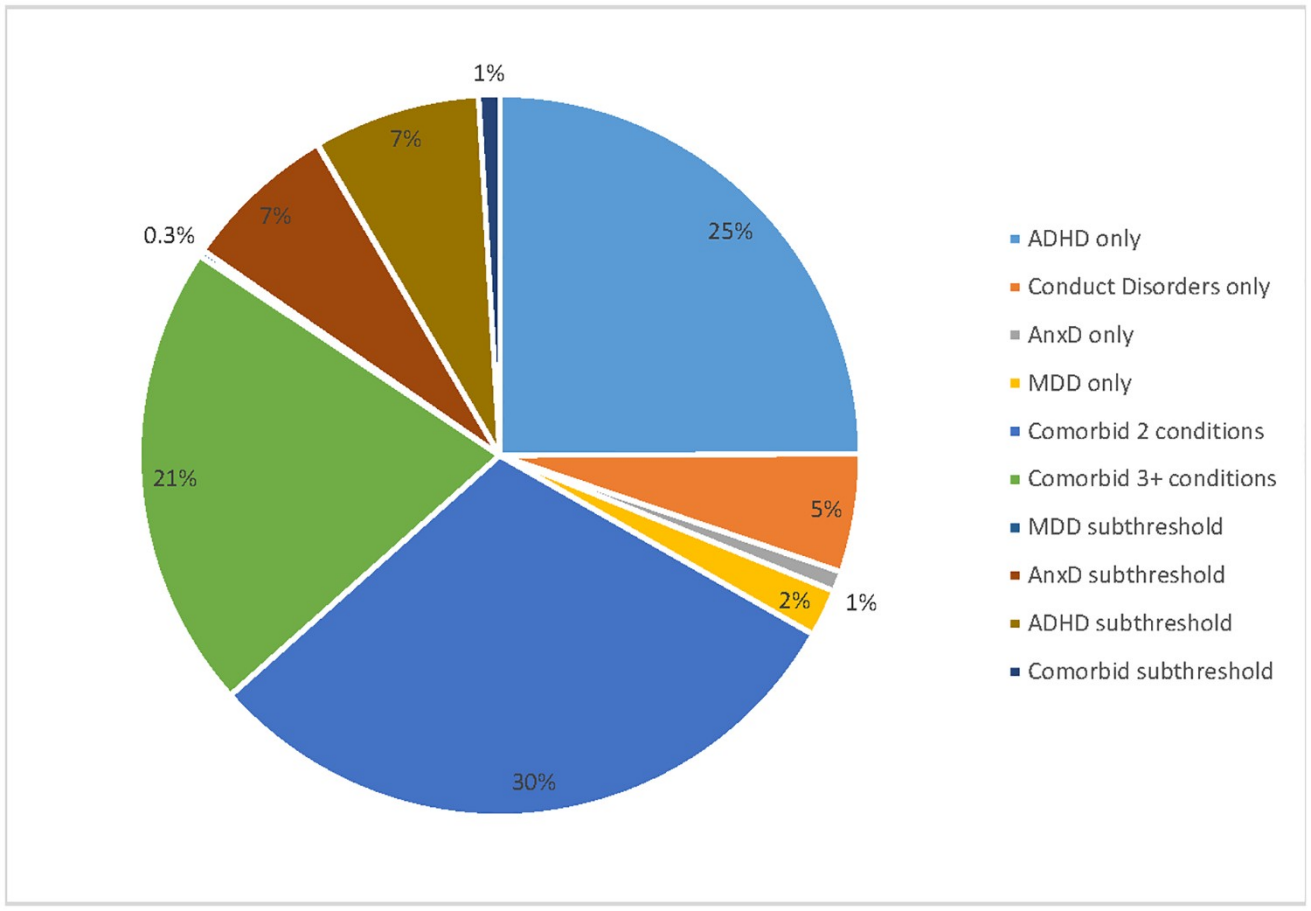
The excess pharmaceutical costs due to metal disorders and subthreshold mental disorders. ADHD: Attention-deficit/hyperactivity disorder, MDD: Major depressive disorder, AnxD Anxiety disorders, CD conduct disorder.

Fig 4 shows the extrapolation of these findings for the entire Australian population (only considering additional costs compared to children/adolescents with no mental disorder) and also taking into account the proportions of children and adolescents in the relevant comparison groups. The estimated adjusted additional Pharmaceutical Benefits Scheme mental health-related cost associated with mental disorders and subthreshold mental disorders for 4 to 17 year olds was AU$68M. The majority of this total additional cost was attributed to ADHD (25%) and the two mental health disorder comorbid group (30%) followed by three plus mental health disorder comorbid group (21%) and subthreshold mental disorders (16%). In subthreshold mental disorders, subthreshold AnxD and subthreshold ADHD contribute 14% of total excess costs. Other single disorders including MDD, AnxD and CD contributed 8% to the total mental health related PBS costs.

Results for the two-part models for Pharmaceutical Benefits Scheme mental and general health related costs is presented in Table 2. Children and adolescents with a mental disorder or subthreshold mental disorder had higher utilization of mental health Pharmaceutical Benefits Scheme compared to those without a mental disorder. Among those who used mental health PBS, the second part of the model indicated that there is a significantly higher expenditure for those with ADHD, CD, with two or three plus comorbid conditions or subthreshold MDD compared to those without a mental disorder. For general health related Pharmaceutical Benefits Scheme cost, only people with AnxD, two comorbid conditions, subthreshold MDD, subthreshold AnxD, and comorbid subthreshold mental disorders were found to have significantly higher use of general health Pharmaceutical Benefits Scheme compared to those without a mental disorder. However, the Pharmaceutical Benefits Scheme costs of general health related medications was significantly greater for those with three comorbid conditions who used Pharmaceutical Benefits Scheme than those without a mental disorder.

**Table 2:** Results for two-part models for total PBS costs.

Disorder categories	Mental health related costs		General health related costs	
	% using health service % (95% CI)	Average cost for those who accessed services $A (95% CI)	% using health service % (95% CI)	Average cost for those who accessed services $A (95% CI)
No mental disorders	0.7 (0.4, 1.1)	197 (87, 308)	25.1 (23.2, 27.0)	154 (83, 226)
Any Mental disorders	15.2 (12.4, 18.0)***	486 (356, 617)***	37.7 (33.8, 41.7)***	113 (72, 155)
ADHD	14.5 (9.3, 19.8)***	752 (428, 1077)**	31.5 (24.7, 38.3)	91 (45, 137)
Conduct disorders	5.2 (0.0, 14.9)	883 (551, 1215)***	28.9 (12.2, 45.6)	84 (21, 146)
Anxiety related disorders	5.1 (1.5, 8.7)*	237 (111, 364)	42.7 (35.3, 50.1)***	113 (48, 179)
Major Depressive Disorders	8.3 (1.8, 14.9)*	414 (0, 900)	29.6 (15.6, 43.5)	148 (18, 279)
Comorbid 2 conditions	24.7 (18.2, 31.1)***	365 (238, 492)*	44.5 (36.5, 52.4)***	142 (53, 231)
Comorbid 3+ conditions	36.4 (19.7, 53.1)***	458 (252, 663)*	37.3 (21.1, 53.4)	63 (8, 118)*
Any Subthreshold disorders	1.8 (1.2, 2.5)**	395 (213, 577)	29.5 (26.9, 32.0)**	129 (90, 168)
Subthreshold MDD	1.4 (0, 4.2)	3201 (1812, 4590)***	37.0 (15.8, 58.1)*	434 (0, 1049)
Subthreshold AnxD	1.0 (0.2, 1.9)	416 (114, 718)	33.0 (28.6, 37.3)**	116 (82, 149)
Subthreshold CD	0	N/A	21.8 (5.0, 38.5)	156 (15, 296)
Subthreshold ADHD	2.2 (0.9, 3.5)*	373 (153, 593)	24.8 (21.1, 28.5)	132 (82, 182)
Subthreshold Comorbid	2.5 (1.2, 3.8)*	184 (93, 275)	32.5 (27.7, 37.3)**	117 (73, 162)

***, statistical significance at the 0.1% level;

**, statistical significance at the 1% level;

*, statistical significance at the 5% level.

Statistically significant results shown in bold.

MDD: Major depressive disorder, AnxD: Anxiety disorders, CD: Conduct disorder, ADHD: Attention-deficit/hyperactivity disorder.

## Discussion

In Australian children and adolescents, MDD, ADHD, CD, and AnxD were associated with a higher use and expenditure in mental health-related Medicare and Pharmaceutical Benefits Scheme services. MDD and having three or more comorbid conditions were associated with the highest mental health-related Medicare costs, while ADHD, CD and subthreshold MDD were associated with the highest mental health related Pharmaceutical Benefits Scheme costs. For those who used Medicare and Pharmaceutical Benefits Scheme services, ADHD was associated with higher mental health-related Pharmaceutical Benefits Scheme costs and general health-related Medicare costs but not mental health-related Medicare costs. This suggests that those with ADHD may seek help from paediatricians or general health related GPs [23]—which were classified as general health related services for the purposes of the current study. Previous research has also found that a large proportion of paediatric consultations have been for behavioural and developmental concerns [24]. Furthermore, given that previous research has also found that pharmaceutical treatment with or without psychotherapy is an effective intervention for children aged 6+ years with ADHD [25], which may explain why ADHD is associated with one-third of the total additional Pharmaceutical Benefits Scheme costs.

Another finding which is somewhat pleasing is that of those children who do access mental health treatment the average number of sessions is greater than published criteria of “minimally adequate treatment” sessions. However, this result should be interpreted with caution given that it is unclear whether the services delivered were evidence-based and disorder specific interventions upon which the ‘minimally adequate treatment’ threshold is based. This is also true of children who have ADHD except they appear to be accessing general health professionals rather than mental health specific professionals. When extrapolating to the population level, the current study found that the additional costs associated with mental disorders and subthreshold mental disorders for 11–17 year olds in Australia was $187M in Medicare claims (including OOP) and $68M for Pharmaceutical Benefits Scheme (including OOP). Findings from this study are difficult to compare with previous studies evaluating the cost of mental disorders as, to our knowledge, this is the first in-depth analysis of economic burden using Australian administrative datasets in this population. Lucas and Bayer Lucas, Bayer (26) reported an additional cost of $27.6M to the Australian Government for mental difficulties (measured by Strengths and Difficulties Questionnaire rather than formal diagnosis of mental disorders) in children from birth up to 8 year-olds. Given that adolescents and young adults aged 12 to 24 years had 8 times higher mental health service use compared to those aged 0 to 11 [24]—our cost estimates may be considered roughly comparable with results reported by Lucas, Bayer [26]. Our study found mental health care related costs of $82M for Medicare and $68M for Pharmaceutical Benefits Scheme. Segal, Guy [24] have found that mental health related Medicare and Pharmaceutical Benefits Scheme costs were respectively $228.2M and $55.1M for children aged 0 to 24 years in 2013–2014. Our extrapolated PBS costs were somewhat higher given that we included anti-hypertensives and anti-convulsants in addition to the more usual mental health related medications as classified by the AIHW and used in the Segal study [24]. The numbers receiving other classes of drugs, including the anti-hypertensives and anti-convulsants were low and it is likely that adding these classes of drugs might have contributed very little to the overall Pharmaceutical Benefits Scheme cost for children and adolescents with mental disorders. Another potential explanation of the differences between our study results and those of Segal is that while the majority of mental health-related Pharmaceutical Benefits Scheme costs can be attributable to mental disorders, mental health-related Medicare services may be used by children who might be dealing with other issues, such as bullying (who may not meet diagnostic criteria for a mental disorder) [27].

Another interesting finding of our study was that the pattern of average OOP costs incurred for both mental health and general health related care appeared to differ according to diagnostic category. In particular, adolescents with MDD incurred higher average mental health related OOP costs compared to those with AnxD. It may be because MDD is common in adolescence but rare in childhood unlike anxiety and is associated with higher functional impairment than anxiety. Furthermore, given that the available effective “talking” or psychological treatments for both MDD and AnxD are relatively similar (CBT) types of therapies, it could be that such children are accessing different types of providers (e.g. children with depression might be more likely to access specialists (including psychiatrists and clinical psychologists) whereas those with anxiety are more likely to be accessing primary care providers and non-specialists for mental health reasons.

The current study also highlighted the economic burden of people with comorbid mental disorders. Findings showed that children and adolescents with multiple disorders incurred higher costs. At a population level, the 30% of children and adolescents with mental disorders who met diagnostic criteria for two or more disorders contributed half of the total mental health-related costs. Importantly, this is not a surprising finding given that comorbidity has been found to result in more serious impairments–and therefore likely to be associated with higher health care costs. For example prior research has found that comorbid mental disorders in Australian adults are associated with greater quality of life decrements [28]. This flags the importance of addressing the needs of those with comorbid mental disorders. These findings of higher costs associated with the occurrence of co-morbid conditions are consistent with what has been found in adults within the Australian context [29,30].

Interestingly, our findings also showed that those with subthreshold mental disorders are large contributors to the total Medicare and Pharmaceutical Benefits Scheme costs especially for those with AnxD or comorbid subthreshold disorders. A probable reason for this is that the prevalence of subthreshold mental disorders was significantly higher than other single mental disorders or comorbid disorders (34.2% vs. <5%) although the average cost per person with subthreshold disorders was significantly lower than the average cost of those with mental disorders. Importantly, this group was associated with significant general health related service costs compared to those with no mental disorders (i.e. AnxD and comorbid subthreshold accounted for 44% of total general health related costs). The possible reason for this is that people with subthreshold mental disorders might seek mental health help from general health professionals such as GPs or paediatricians rather than mental health professionals as they have not met the criteria for a mental health diagnosis. Previous research showed that adolescents with subthreshold mental disorders also require intensive interventions provided over a long time period in order to achieve recovery as those with mental disorders [31]. Further research is required to investigate illness trajectories of this group to design appropriate and cost-effective access to care for this under-researched group.

### Strengths and limitations

The strengths of this study include that the two administrative data sets of Medicare and Pharmaceutical Benefits Scheme claims data provide much greater accuracy than other techniques of assessing costs, such as resource use questionnaires. Furthermore, we were able to link this data to comprehensively assessed diagnostic data via the YMM survey, thus overcoming the difficulties of analysing MBS and PBS data in isolation.

However, the costs reported here are an underestimate of the full economic burden of mental disorders. Medicare and Pharmaceutical Benefits Scheme data does not include the cost of the large number of mental health treatment and prevention services provided by non-government organisations, school counsellors or state based child and adolescent mental health services. Other costs such as the cost of hospital admissions and emergency department presentations are also not included in such Medicare and Pharmaceutical Benefits Scheme data. Similarly, any medications or supplements that are not covered under the Pharmaceutical Benefits Scheme were not included in this study. Further research is required to explore the full picture of what services and costs, in terms of content, are being used by adolescents with mental disorders.

Although the use of the two-part model for the large number of zeros may improve the fit of the statistical models, the small sample size of those with positive values may have prevented detection of statistically significant differences between groups [21].

While it is clear that additional health care expenditure is associated with mental health diagnoses in children and adolescents, this data does not provide any indication of the benefit or outcome from these services. We are therefore unable to determine whether these costs are associated with cost-effective service provision in terms of treatment content of MBS services in particular [32].

Finally whether this amount of Medicare and Pharmaceutical Benefits Scheme costs is too high or not high enough remains to be determined as the “value” of this spending cannot be easily determined from such administrative data sets. However, given the YMM survey has also found that 44% of children who meet criteria for mental health disorders (including severe disorders) had not accessed any form of treatment suggests that the Medicare and Pharmaceutical Benefits Scheme costs should probably be even higher to meet unmet need [22,33]. Furthermore, the cost estimates for mental disorders and subthreshold mental disorders will be valuable for use in future economic evaluations of interventions for prevention or treatment of mental disorders.

## Conclusion

Mental health diagnoses and symptoms were associated with $234 million in annual incremental health care costs for medical services and medications in Australian children and adolescents aged 4–17 years old. Approximately sixteen percent of this cost is in the form of OOP payments. The current analysis only represents a portion of the total health care cost since it was limited to Medicare and Pharmaceutical Benefits Scheme administrative claims data. Further research is needed to estimate the full picture of economic burden of mental disorders in children and adolescents.

## Supporting information

S1 Fig Overview of consent and number of sample size for Medicare-funded medical and pharmaceutical services. (TIF)

## References

[1] Olesen J, Gustavsson A, Svensson M, Wittchen HU, Jönsson B, Group CS, et al. The economic cost of brain disorders in Europe. Eur J Neurol. 2012;19(1):155–62. 10.1111/j.1468-1331.2011.03590.x 22175760

[2] Fatori D, Salum G, Itria A, Pan P, Alvarenga P, Rohde LA, et al. The economic impact of subthreshold and clinical childhood mental disorders. J Ment Health. 2018;27(6):588–94. 10.1080/09638237.2018.1466041 29708045

[3] Beecham J. Annual research review: Child and adolescent mental health interventions: A review of progress in economic studies across different disorders. J Child Psychol Psychiatry. 2014;55(6):714–32. 10.1111/jcpp.12216 24580503 PMC4657502

[4] Lawrence D, Johnson S, Hafekost J, Boterhoven de Haan K, Sawyer M, Ainley J, et al. The mental health of children and adolescents: Report on the second Australian Child and Adolescent Survey of Mental Health and Wellbeing. 2015.

[5] Department of Human Services. Medicare benefits schedule item statistics. Canberra: Medicare benefits schedule item statistics; 2020.

[6] Department of Human Services. Pharmaceutical benefits schedule item statistics Canberra2020. http://medicarestatistics.humanservices.gov.au/statistics/pbs_item.jsp.

[7] Knapp M, Wong G. Economics and mental health: the current scenario. World Psychiatry. 2020;19(1):3–14. 10.1002/wps.20692 31922693 PMC6953559

[8] König H, König H-H, Konnopka A. The excess costs of depression: a systematic review and meta-analysis. Epidemiol Psychiatr Sci. 2020;29. 10.1017/S2045796019000180 PMC8061284 30947759

[9] Konnopka A, König H. Economic burden of anxiety disorders: a systematic review and meta-analysis. Pharmacoeconomics. 2020;38(1):25–37. 10.1007/s40273-019-00849-7 31646432

[10] Glick HA, Doshi JA, Sonnad SS, Polsky D. Economic evaluation in clinical trials: OUP Oxford; 2014.

[11] Hafekost J, Lawrence D, Boterhoven de Haan K, Johnson SE, Saw S, Buckingham WJ, et al. Methodology of young minds matter: The second Australian child and adolescent survey of mental health and wellbeing. Aust N Z J Psychiatry. 2016;50(9):866–75. 10.1177/0004867415622270 26698821

[12] Shaffer D, Fisher P, Lucas CP, Dulcan MK, Schwab-Stone ME. NIMH Diagnostic Interview Schedule for Children Version IV (NIMH DISC-IV): description, differences from previous versions, and reliability of some common diagnoses. J Am Acad Child Adolesc Psychiatry. 2000;39(1):28–38. 10.1097/00004583-200001000-00014 10638065

[13] Health CDo, Care A. The Australian health care system: An outline. Australian Institute of Health and Welfare Canberra; 2000.

[14] Britt H, Miller GC, Bayram C, Henderson J, Valenti L, Harrison C, et al. A decade of Australian general practice activity 2006–07 to 2015–16: Sydney University Press; 2016.

[15] World Health Organization. ATC-Structure and principles. WHO Collaborating Centre for Drug Statistics Methodology; 2018.

[16] Australian Institute of Health. Mental Health Services: In Brief 2019: Australian Institute of Health; 2019.

[17] Arnsten AF, Scahill L, Findling RL. Alpha-2 adrenergic receptor agonists for the treatment of attention-deficit/hyperactivity disorder: emerging concepts from new data. J Child Adolesc Psychopharmacol. 2007;17(4):393–406. 10.1089/cap.2006.0098 17822336

[18] Davidson J. Pharmacotherapy of social anxiety disorder: what does the evidence tell us? J Clin Psychiatry. 2006;67:20–6. 17092192

[19] Australian Bureau of Statistics. Australian demographic statistics—3101.0. Australian Bureau of Statistics, 2019.

[20] Ramsey SD, Willke RJ, Glick H, Reed SD, Augustovski F, Jonsson B, et al. Cost-effectiveness analysis alongside clinical trials II—an ISPOR Good Research Practices Task Force report. Value Health. 2015;18(2):161–72. 10.1016/j.jval.2015.02.001 25773551

[21] Deb P, Norton EC. Modeling Health Care Expenditures and Use. Annu Rev Public Health. 2018;39(1):489–505. 10.1146/annurev-publhealth-040617-013517 . 29328879

[22] Lawrence D, Hafekost J, Johnson SE, Saw S, Buckingham WJ, Sawyer MG, et al. Key findings from the second Australian child and Adolescent Survey of Mental Health and Wellbeing. Aust N Z J Psychiatry. 2016;50(9):876–86. 10.1177/0004867415617836 26644606

[23] Sciberras E, Lucas N, Efron D, Gold L, Hiscock H, Nicholson JM. Health care costs associated with parent-reported ADHD: a longitudinal Australian population–based study. J Atten Disord. 2017;21(13):1063–72. 10.1177/1087054713491494 23816972

[24] Segal L, Guy S, Furber G. What is the current level of mental health service delivery and expenditure on infants, children, adolescents, and young people in Australia? Aust N Z J Psychiatry. 2018;52(2):163–72. 10.1177/0004867417717796 28709383

[25] Catala-Lopez F, Hutton B, Núñez-Beltrán A, Page MJ, Ridao M, Saint-Gerons DM, et al. The pharmacological and non-pharmacological treatment of attention deficit hyperactivity disorder in children and adolescents: a systematic review with network meta-analyses of randomised trials. PloS one. 2017;12(7):e0180355. 10.1371/journal.pone.0180355 28700715 PMC5507500

[26] Lucas N, Bayer JK, Gold L, Mensah FK, Canterford L, Wake M, et al. The cost of healthcare for children with mental health difficulties. Aust N Z J Psychiatry. 2013;47(9):849–58. 10.1177/0004867413491152 23719183

[27] Evans-Lacko S, Takizawa R, Brimblecombe N, King D, Knapp M, Maughan B, et al. Childhood bullying victimization is associated with use of mental health services over five decades: a longitudinal nationally representative cohort study. Psychol Med. 2017;47(1):127–35. 10.1017/S0033291716001719 27677437

[28] Mihalopoulos C, Engel L, Le LK-D, Magnus A, Harris M, Chatterton ML. Health state utility values of high prevalence mental disorders in Australia: results from the National Survey of Mental Health and Wellbeing. Qual Life Res. 2018;27(7):1815–25. 10.1007/s11136-018-1843-2 29633165

[29] Teesson M, Slade T, Mills K. Comorbidity in Australia: findings of the 2007 national survey of mental health and wellbeing. Aust N Z J Psychiatry. 2009;43(7):606–14. 10.1080/00048670902970908 19530017

[30] Lee Y-C, Chatterton ML, Magnus A, Mohebbi M, Le LK-D, Mihalopoulos C. Cost of high prevalence mental disorders: findings from the 2007 Australian National Survey of mental health and wellbeing. Aust N Z J Psychiatry. 2017;51(12):1198–211. 10.1177/0004867417710730 28565923

[31] Cross SP, Hermens DF, Hickie IB. Treatment patterns and short-term outcomes in an early intervention youth mental health service. Early intervention in psychiatry. 2016;10(1):88–97. 10.1111/eip.12191 25263607

[32] Harris MG, Hobbs MJ, Burgess PM, Pirkis JE, Diminic S, Siskind DJ, et al. Frequency and quality of mental health treatment for affective and anxiety disorders among Australian adults. Med J Aust. 2015;202(4):185–9. 10.5694/mja14.00297 25716600

[33] Sawyer MG, Reece CE, Sawyer AC, Johnson SE, Hiscock H, Lawrence D. Access to health professionals by children and adolescents with mental disorders: Are we meeting their needs? Aust N Z J Psychiatry. 2018;52(10):972–82. 10.1177/0004867418760713 29498290

