# Supplementary figures and images for: The cost of Medicare-funded medical and pharmaceutical services for mental disorders in children and adolescents in Australia
Source: PLoS One. 2021 Apr 9;16(4):e0249902. doi: 10.1371/journal.pone.0249902 (PMC8034743; doi:10.1371/journal.pone.0249902)

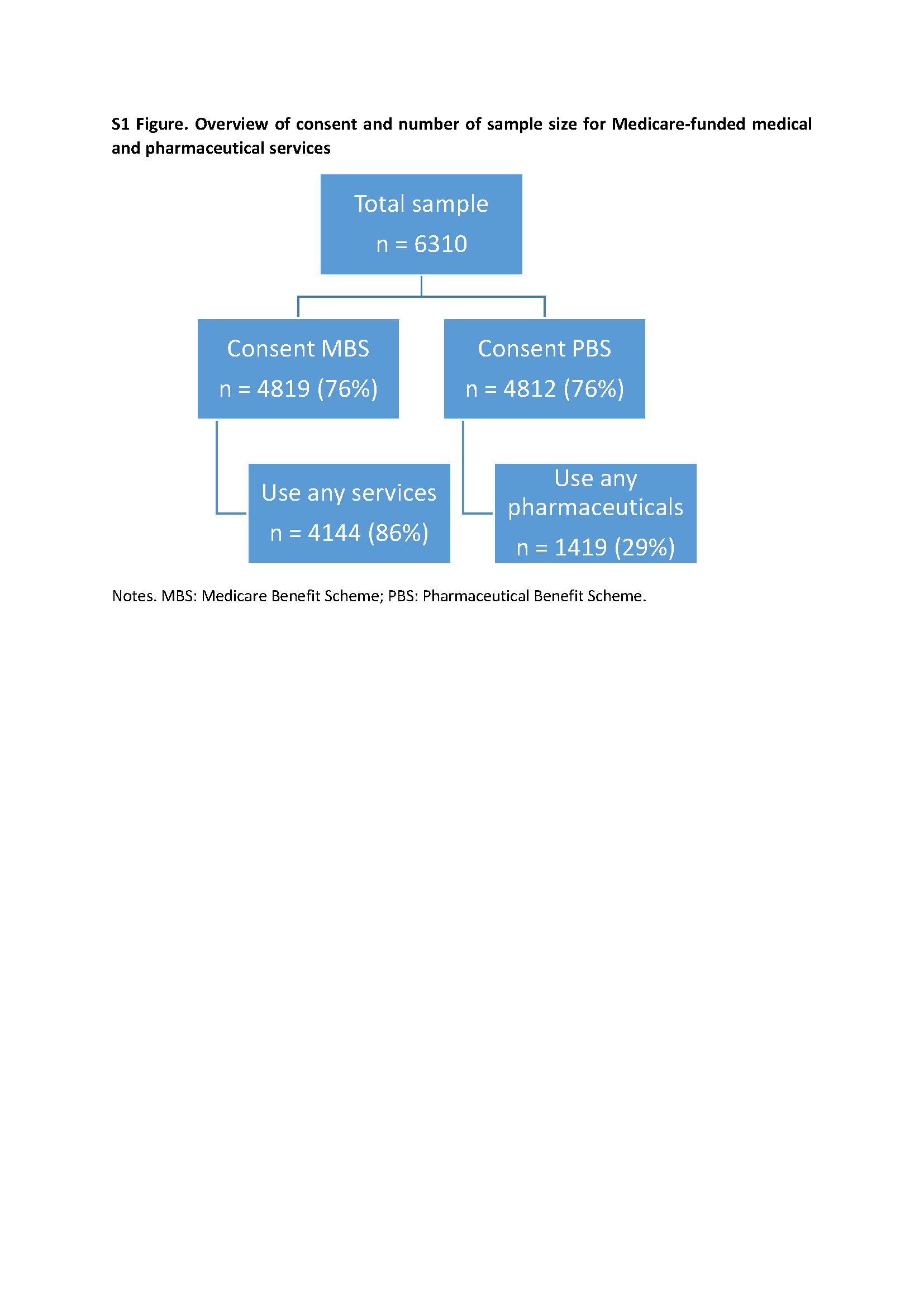

Supplement: S1 Fig — (TIF) [file pone.0249902.s001.tif]
